# The Neural Mechanisms of Cognitive Control in the Category Induction Task

**DOI:** 10.3389/fpsyg.2022.743178

**Published:** 2022-02-15

**Authors:** Xueli Cai, Guo Li, Qinxia Liu, Feng Xiao, Youxue Zhang, Yifeng Wang

**Affiliations:** ^1^Psychological Research and Counseling Center, Southwest Jiaotong University, Chengdu, China; ^2^Department of Education Science, Innovation Center for Fundamental Education Quality Enhancement of Shanxi Province, Shanxi Normal University, Linfen, China; ^3^School of Education and Psychology, Chengdu Normal University, Chengdu, China; ^4^Institute of Brain and Psychological Sciences, Sichuan Normal University, Chengdu, China

**Keywords:** cognitive control, conflict information, dACC, vACC, category induction

## Abstract

According to the conflict monitoring hypothesis, conflict monitoring and inhibitory control in cognitive control mainly cause activity in the anterior cingulate cortex (ACC) and control-related prefrontal cortex (PFC) in many cognitive tasks. However, the role of brain regions in the default mode network (DMN) in cognitive control during category induction tasks is unclear. To test the role of the ACC, PFC, and subregions of the DMN elicited by cognitive control during category induction, a modified category induction task was performed using simultaneous fMRI scanning. The results showed that the left middle frontal gyrus (BA9) and bilateral dorsal ACC/medial frontal gyrus (BA8/32) were sensitive to whether conflict information (with/without) appears, but not to the level of conflict. In addition, the bilateral ventral ACC (BA32), especially the right vACC, a part of the DMN, showed significant deactivation with an increase in cognitive effort depending on working memory. These findings not only offer further evidence for the important role of the dorsolateral PFC and dorsal ACC in cognitive control during categorization but also support the functional distinction of the dorsal/ventral ACC in the category induction task.

## Introduction

Cognitive control is the ability to guide thoughts and actions in accordance with internal goals (Miller and Cohen, 2001). Cognitive control is involved in many important mental processes such as attention, working memory (WM), decision-making, and planning (Nyberg, 2018; Chen, 2019). There are two differentiated processes in cognitive control: (a) monitoring (i.e., the ability to detect and evaluate abnormal situations such as conflicts and errors, and then use the control function to intervene in these situations) and (b) inhibition control (i.e., the ability to ignore and inhibit the processing of irrelevant information) (Miyake et al., 2000; Chen, 2019). The conflict monitoring hypothesis assumes that the conflict monitoring system first evaluates current levels of conflict and then passes this information to the centers responsible for control, triggering them to adjust the strength of their influence on processing (Botvinick et al., 2001, 2004). Conflict monitoring and control mainly involve the anterior cingulate cortex (ACC) and prefrontal cortex (PFC). The ACC monitors the occurrence of conflict information, whereas the PFC participates in inhibition control and makes appropriate behavioral responses (Botvinick et al., 2001; Veen and Carter, 2002; Yeung, 2013; Nyberg, 2018).

The impact of domain-general cognitive control abilities on category induction has also gained increasing research interest (Bigman and Pratt, 2004; Chen et al., 2008; Garcin et al., 2012; Cai et al., 2014; Gao et al., 2016). The basic process of category induction is comparing similarities (detecting congruency) and differences (inhibiting incongruency), abstracting relationships among stimuli, and forming categories (Bigman and Pratt, 2004). Chen et al. (2008) used a partially incongruent category induction task to test the conflict monitoring hypothesis; both conflict detection and conflict control need to be elicited in one trial, and conflict detection should take place before conflict control. The study found that the ACC responded to the process of identifying conflicting features and the PFC controlled this conflict in category decisions. However, the limited spatial resolution of Event-related potential (ERP) in the task reduces the accuracy of the position of the ACC and PFC. Garcin et al. (2012) demonstrated that similarity detection and dissimilarity inhibition involve the anterior ventrolateral PFC (VLPFC) bilaterally, with right–left asymmetry. Cai et al. (2014) and Gao et al. (2016) detected congruent features and inhibited incongruent features within a category using three-stepwise category induction with fMRI. Two studies found that stronger activations in the middle and mid-ventrolateral PFC, bilateral parietal cortex, and putamen were associated with the processes of detecting congruency and inhibiting incongruent features. In summary, all these studies highlighted the different roles of the ACC, PFC, and other regions in the cognitive control of category induction, but no attempt was made to discover in detail the functions of these regions (such as the dorsal and ventral ACC) in the cognitive control of category induction. Previous studies have proposed that an increase in functional antagonism (i.e., anticorrelation) between activities in the control network and the default mode network (DMN) as a function of increased task demands is critical for optimal cognitive performance (Kelly et al., 2008; Anticevic et al., 2012). In other words, brain regions in the DMN are routinely deactivated during cognitive tasks such as WM and classification tasks (Greicius et al., 2003; Hampson et al., 2006; Mayer et al., 2010; Wei et al., 2013; Ceko et al., 2015; Finc et al., 2017; Breukelaar et al., 2020). Whether some default mode regions (e.g., the vACC) also participate in the cognitive process of the category induction task warrants further exploration.

**FIGURE 1:**
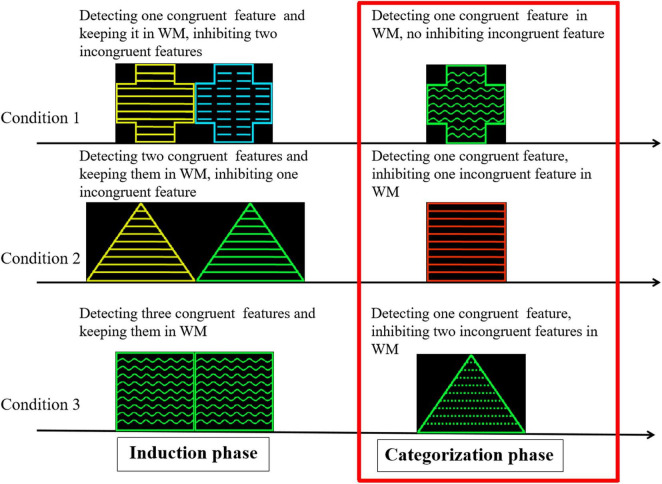
Cognitive analysis in induction and categorization phase in category induction task. Stimuli samples were geometric figures that varied along three perceptual dimensions, with four attributes for each dimension: shape (triangle, square, round, and cross), color (yellow, blue, green, and red), and stripe direction (wavy, streaked, dotted, and straight lines).

The purpose of this study was to confirm the role of the ACC and PFC in the cognitive control process of the category induction task while exploring how deactivation of the subregions (mostly the vACC) of the DMN changes with increasing cognitive load. A modified category induction task was used in accordance with previous studies (Bigman and Pratt, 2004; Chen et al., 2008). The task consisted of an induction phase and a categorization phase. Two geometric figures are presented simultaneously during the induction phase. Participants were required to form a category in each induction phase trial. This study differed from the previous research paradigm in the following two aspects. First, the experiment rule was that the category was defined by one feature in each trial. However, one definite category could not be formed in two of the three conditions (i.e., there were two or three possible categories) based on the experimental design. Second, when one feature of the probe was congruent with only one feature of one definite category, the probe was regarded as a member of the category during categorization. When one feature of the probe was congruent with one of the features in two or three possible categories (concepts), it was difficult for participants to judge whether the probe was regarded as a member of the categories and elicit a conflict categorization explanation during categorization. In the latter case, there are two or three possible categories. These congruent and incongruent features were processed simultaneously in the latter two cases during categorization. Therefore, the number of features in the possible concepts determines whether conflict occurrence and control are involved in categorization. The congruent features elicited a matching and positive categorization process under the premise of one definite concept, but the incongruent features elicited a conflict process and reduced the strength of positive categorization under the premise of non-unique concepts. Participants detected only one congruent feature in all trials, but the number of inhibiting incongruent features varied between zero, one, and two during categorization. Thus, the neural activation of cognitive control can be examined by comparing the categorization process among the different conditions in the present study (Figure 1).

Many studies supporting the conflict-monitoring hypothesis also confirm that control is recruited following detection in the medial PFC (including BA 6, 8, 24, 32) of competition or conflict in information processing. The medial PFC, particularly the dorsal ACC (mainly responsible for cognitive function), monitors conflicts in information processing and recruits the dorsolateral PFC (DLPFC) to resolve competition as needed (Ridderinkhof et al., 2004; Carter and Veen, 2007; McNab and Klingberg, 2008; Freedman and Assad, 2011; Yeung, 2013; Heilbronner and Hayden, 2016; Berry et al., 2017; Davis et al., 2017; Nyberg, 2018). In addition, as one of the two subdivisions in the ACC, the ventral anterior cingulate cortex (vACC) subserves the affective process (Bush et al., 2000; Grinband et al., 2011; Lockwood and Wittmann, 2018) and monitors emotional conflict (Shapira-Lichter et al., 2018). Other functional imaging studies have found that the deactivation of vACC belonging to the DMN were positively correlated with cognitive load and increasing task complexity (Greicius et al., 2003; Hampson et al., 2006; Mayer et al., 2010; Anticevic et al., 2012; Wei et al., 2013; Hearne et al., 2015).

In this study, the core process was the cognitive control of extracting congruent features and inhibiting incongruent features under the involvement of WM. According to the conflict-monitoring hypothesis (Botvinick et al., 2001, 2004), we hypothesized that activation in the dACC and DLPFC (mainly referring to the middle frontal gyrus) may be responsible for monitoring the appearance of conflicting information and inhibition control (ignoring and inhibiting incongruent/irrelevant information), and deactivation of the vACC in the DMN may be found with increasing cognitive effort depending on WM (Greicius et al., 2003; Hampson et al., 2006; Mayer et al., 2010; Wei et al., 2013; Ceko et al., 2015; Finc et al., 2017; Buckner and DiNicola, 2019; Breukelaar et al., 2020).

## Materials and Methods

### Participants

Twenty right-handed healthy university students with normal or corrected-to-normal vision were paid to participate in the experiment (male/female: 10/10; mean age: 22 years; age range: 19–23 years). The mean educational level was 15.9 years (*SD* = 1.71; range 13–19). All participants met the criteria for MRI scans (i.e., no metallic implants, no claustrophobia, and a head size compatible with the custom head coil). In addition, the participants had no known neurological or psychiatric injuries or disorders and were not taking any psychoactive medications or drugs. Data from four participants were excluded before analysis because of unacceptable head motion or poor performance in the experimental tasks. All participants provided written informed consent before the scan session. This study was approved by the ethical review board of the Faculty of Psychology at the Southwest University.

### Materials and Tasks

Stimuli were geometric figures that varied along three perceptual dimensions, with four attributes for each dimension: shape (triangle, square, round, and cross), color (yellow [225, 235, 0], blue [0, 221, 255], green [0, 255, 50], and red [255, 51, 0]), and stripe direction (wavy, streaked, dotted, and straight lines). The sizes of the figures were approximately 5.92 cm in height for triangles, 4.28 cm in width and height for squares, 4.28 cm in diameter for circles, and 4.28 cm in wheelbase for crosses.

Each trial consisted of three phases. (1) Induction: Two geometric figures (S1 and S2) were simultaneously presented. Participants were instructed to extract congruent features associated with the target category in each trial and keep them in mind to solve the subsequent categorization (Figure 1). (2) Categorization: A probe figure (S3) was presented. In cognitive processing, participants needed to compare the probe feature with the preceding features stored in WM. Hence, WM was involved in the categorization process. In this phase, the participants were instructed to determine whether the probe was a member of the category defined by S1 and S2. Participants were asked to respond by pressing one button for positive responses and another for negative responses. (3) Feedback: Feedback was provided to indicate whether participants’ responses were correct or incorrect.

Three categorization conditions were designed according to the number of congruent features (one, two, or three) during the induction phase. In each trial, the final target category was defined using only one attribute to induce different conditions.

### Experimental Conditions

**Condition 1 (C1):** In the induction phase, S1 and S2 shared one congruent feature. In the categorization phase, the probe shared one congruent feature with the preceding stimulus. Under this condition, the probability of the probe being a member of the target category is 100% (Figure 1).

**Condition 2 (C2):** In the induction phase, S1 and S2 shared two congruent features. In the categorization phase, the probe shared one of the two congruent features of the preceding stimuli. In this instance, the probability of the probe being a member of the target category is 50%.

**Condition 3 (C3):** In the induction phase, S1 and S2 were the same. In the categorization phase, the probe shared one of three congruent features of the preceding stimuli. The probability of the probe being a member of the target category is 33.3%.

To balance the impact of expectations on research, task-set control conditions were used. When the probes did not share congruent features with the preceding stimuli in each condition, the trials were used as controls. In all trials, congruent features were assigned randomly such that incongruent features could not be predicted.

### Procedure

**FIGURE 2:**
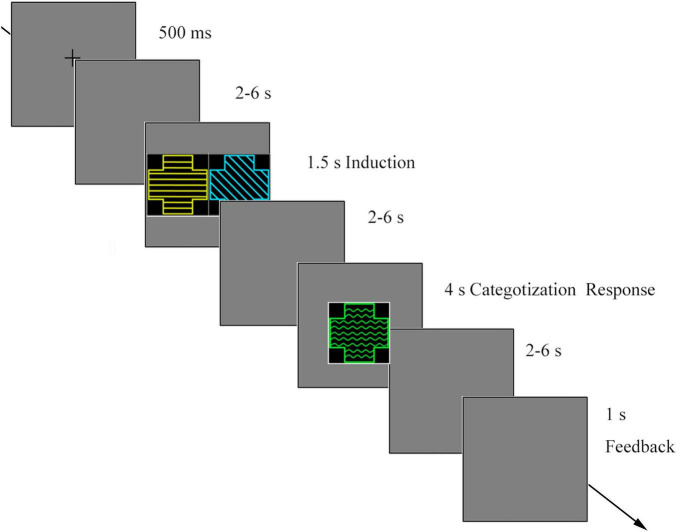
The procedure of the category induction task.

At the beginning of each trial, a fixation cross appeared on the screen for 500 ms. After a random blank screen was presented for 2–6 s, S1 and S2 were simultaneously presented for 1.5 s. After a random blank screen (2–6 s), a probe was presented for a maximum of 4 s or until the participants responded. Participants were asked to determine whether the probe shared the same category membership with the preceding two stimuli. After a blank screen (2–6 s), feedback was presented for participants’ categorization for 1 s (Figure 2).

The participants completed five runs, each lasting 11 min. The C1 consisted of 30 trials. C2 and C3 each consisted of 60 trials. The control condition comprised 24 trials. There were 174 trials in total. The first four runs consisted of 35 trials and the last run consisted of 34 trials. Each run contained all the categorization types. All trials were presented in a randomized order.

Before the formal experiment, the researcher informed the participants of the task instructions in detail, and sufficient practice was provided to ensure that participants understood the task.

### fMRI Data Acquisition

fMRI data acquisition was performed using a Siemens TRIO 3.0 T full-body MRI scanner (Siemens, Erlangen, Germany). For each participant, anatomical images (256 × 256 × 176) with 1 × 1 × 1 mm resolution were obtained using a T1-weighted three-dimensional magnetization prepared rapid gradient echo (MPRAGE) sequence (inversion time, 900 ms; repetition time, 1,900 ms; echo time, 2.52 ms; flip angle, 9°). Functional scanning used echo planar imaging (EPI) sequence (flip angle 90°; TR, 2,000 ms; TE, 30 ms; FoV, 192 × 192 mm^2^; matrix size, 64 × 64; interslice skip, 0.99 mm; voxel size, 3 × 3 × 3 mm^3^; slices, 32) with prospective acquisition correction (PACE). This helped to reduce its impact on data acquisition. The slices were positioned along the anterior commissure-posterior commissure plane.

### fMRI Data Analyses

The data were analyzed using SPM8 to preprocess the functional images (Friston et al., 1997). Slice timing was used to correct the slice order. The data were realigned to estimate and modify the six parameters of head movement, and the first three images were discarded to achieve magnet-steady images. These images were then normalized to the MNI space in 3 × 3 × 3 mm^3^ voxel sizes. Normalized data were spatially smoothed with a Gaussian kernel. The full width at half maximum (FWHM) was specified as 8 × 8 × 8 mm^3^. After preprocessing, nine regressors from each run (induction, categorization, and feedback phases among the three conditions) were modeled to create the design matrix. For each participant, all five runs were modeled in a general linear model (GLM). They were convolved with the canonical hemodynamic response function, and the six realignment parameters for each participant were included as confounding factors.

We directly examined brain activity in the DLPFC, mPFC/dACC, and vACC during categorization to explore the cognitive control process by analyzing regions of interest (ROIs). Five regions of interest (ROIs) were defined as regions where previous studies demonstrated activation associated with similarity detection, similarity-based categorization, and cognitive control (Botvinick et al., 2001; Garcin et al., 2012; Yeung, 2013; Gao et al., 2016; Davis et al., 2017; Nyberg, 2018): the left middle frontal gyrus (BA9; MNI coordinates:–50, 14, 32) (Gao et al., 2016; Nyberg, 2018), bilateral medial frontal gyrus/dACC (BA8/32; MNI coordinate: left: –6, 21, 45; right: 6, 21, 45), and bilateral ventral anterior cingulate (BA32; MNI coordinate: left: –9, 30, –6; right: 9, 30, –6) (Botvinick et al., 2001; Greicius et al., 2003; Mayer et al., 2010; Yeung, 2013). The ROIs were constructed by creating a sphere with a radius of 8 mm around the centers defined by the aforementioned sets of coordinates. The mean ROI data were obtained for each original event type from the first-level analysis of each participant. Beta values from all ROIs were subjected to one-way analysis of variance.

## Results

### Behavioral Results

We investigated accuracy rates (ACC) and reaction times (RT) during the categorization phase of participants who selected positive categorization (shared category membership) in the three category induction conditions. The control conditions (no shared features with the preceding induction stimuli in the three conditions) were not analyzed as a small number of trials.

As shown in Table 1, the accuracy rates (mean ± *SD*) for C1, C2, and C3 were 90, 49, and 48%, respectively. A one-way ANOVA revealed significant differences among the three conditions [*F*_(2, 45)_ = 120.09, *p* < 0.001, partial η^2^ = 0.84]. *Post-hoc* tests indicated that accuracies were significantly higher under C1 than under C2 and C3 (*p*s < 0.001). No significant difference in accuracy was observed between C2 and C3 (*p* = 0.70).

**TABLE 1:** Accuracy rates (ACC) and reaction times (RT) across three conditions.

Condition	Accuracy	RT (ms)
C1	0.90 ± 0.12	1194.12 ± 317.54
C2	0.49 ± 0.05	1562.51 ± 292.81
C3	0.48 ± 0.06	1702.23 ± 285.85

*Mean and standard deviation (M ± SD).*

A one-way ANOVA revealed significant differences among the three conditions for RTs [*F*_(2, 45)_ = 10.95, *p* < 0.001, partial η^2^ = 0.33]. *Post-hoc* tests revealed that the RTs for C1 were significantly shorter than those for C2 and C3 (*p*s < 0.001). However, the RT for C2 was not significantly shorter than that for C3 (*p* = 0.17; Table 1).

### The fMRI Results of Categorization

We analyzed the brain activation for positive categorization during the categorization phase. The mean number of valid trials for each participant in the three conditions was 27 for C1, 38 for C2, and 31 for C3.

**FIGURE 3:**
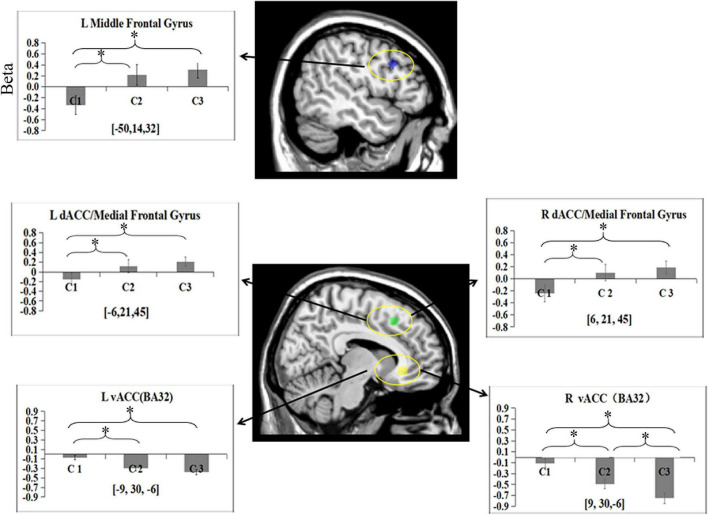
Beta means within five ROIs for categorization phase among three conditions. Error bars represent SE of the mean across all participants. L, left; R, right. *p < 0.05.

To test the activation of the DLPFC, mPFC/dACC, and vACC related to cognitive control during categorization, the data of the five ROIs were subjected to a one-way ANOVA separately. The main effect of the condition was found in the left middle frontal gyrus [BA9; *F*_(2, 45)_ = 4.14, *p* = 0.02, partial η^2^ = 0.16]. *Post hoc* analysis revealed that there were more significant activations in C2 (*M* = 0.22) and C3 (*M* = 0.31) in the area than in C1 (*M* = –0.33; *p*s < 0.05). There was no significant difference in activity between C2 and C3 (*p* > 0.05) (Figure 3).

One-way ANOVA revealed a main effect of condition in the bilateral medial frontal gyrus/dACC (BA8/32) [left: *F*_(2, 45)_ = 2.73, *p* = 0.08, partial η^2^ = 0.11; right: *F*_(2, 45)_ = 3.14, *p* = 0.05, partial η^2^ = 0.12]. *Post hoc* analysis revealed that more activities in these two ROIs were found in C3 (left: *M* = 0.24; right: *M* = 0.19) than in C1 (left: *M* = –0.16; right: *M* = –0.24; *p*s < 0.05). More activities were found in these two ROIs in C2 (left *M* = 0.12; right *M* = 0.11) than in C1 (*p*s < 0.05). No differences were observed in the two ROIs between C2 and C3 (*p*s > 0.05).

One-way ANOVA revealed a main effect of condition in the bilateral ventral anterior cingulate cortex (vACC) (BA32) [left: *F*_(2, 45)_ = 8.5, *p* = 0.001, partial η^2^ = 0.28; right: *F*_(2, 45)_ = 14.18, *p* = 0.000, partial η^2^ = 0.39]. *Post hoc* analysis showed more deactivation in the bilateral vACC for C2 (left: *M* = –0.29; right: *M* = –0.49) and C3 (left: *M* = –0.38; right: *M* = –0.75) than for C1 (left: *M* = –0.08; right: *M* = –0.11) (*p*s < 0.05). No significant difference was observed in the left vACC between C2 and C3 (*p* > 0.05). However, more significant deactivation was observed in the right vACC in C3 than in C2 (*p* < 0.05).

## Discussion

The study focused on the activation associated with cognitive control for incongruent features when forming a category during the process of categorization. Behavioral data revealed longer RTs and lower ACC rates for C2 and C3 than for C1, reflecting higher cognitive resources required in the first two conditions. Two or three possible categories (concepts) were formed in C2 and C3 during the induction process. This could have led to the participants’ conflict categorization, and categorization strengthening was weaker in these two conditions than in C1.

Our fMRI data indicated that the left middle frontal gyrus (BA9) and bilateral dorsal ACC (dACC)/medial frontal gyrus (medial FG) (BA8/32) showed increased positive activity from C1 to C3, which is in accordance with our behavioral findings. Similar to the cognitive process of Chen et al.’s (2008) study, only one congruent feature was extracted, and no incongruent feature was inhibited in WM for category C1. One congruent feature was extracted and one incongruent feature was inhibited in the WM for category in C2. One congruent feature was extracted, and two incongruent features were inhibited in WM for category in C3. Hence, there is a one-unit WM load difference and a crucial difference in conflict (with/without) between C1 and C2. There was also a one-unit WM load difference and a difference in conflict (level difference) between C2 and C3. These findings indicate that conflict (with/without) may be the main factor rather than the WM load difference. Many human neuroimaging studies have emphasized that cognitive control activates the DLPFC and posterior medial frontal cortex (pMFC) (Botvinick et al., 2004; Carter and Veen, 2007; Berry et al., 2017; Nyberg, 2018). The posterior medial frontal cortex (pMFC), including the dorsal ACC, along with other brain structures, plays a general role in coding unfavorable outcomes, response errors, response conflict, and decision uncertainty (Ridderinkhof et al., 2004; Seger et al., 2015; Wu et al., 2020). The conflict-monitoring hypothesis emphasizes that DLPFC is involved in the implementation of control processes to resolve conflicts (Berry et al., 2017; Nyberg, 2018). The dorsal ACC (dACC) is specialized for the detection of environmental conditions, signaling the need for the implementation of cognitive control and being responsible for sending “triggers” to other systems specialized in the actual implementation of control (Botvinick et al., 2004; Carter and Veen, 2007). This suggests that the left middle frontal gyrus (BA9) and dACC/medial frontal gyrus (BA8/32) were sensitive to whether incongruent information appears, but not to the levels of incongruent information in this study. The results of the present study may provide direct support for the conflict-monitoring hypothesis of cognitive control (Botvinick et al., 2001, 2004).

Although participants can fully understand that a probe stimulus matches one feature of the pair in the present study, it is still uncertain whether they will get it correct because identifying the match only achieves correct feedback the probability of 50 and 33.3% in C2 and C3, respectively. This makes it much more like “known” uncertainty about the outcome of a probabilistic process than a decisional uncertainty problem. When there was one congruent feature between the probe and possible categories in WM in C2 and C3, participants might have been more hesitant to make choices in C2 and C3 than in C1. In other words, participants may have been prone to producing response conflicts in C2 and C3. In this study, the DPLFC-dACC/medial FG may reflect two types of conflict. One was the existence of task-related representational conflicts (detecting incongruent features), and the other was conflict at the response level (category uncertainty) (Ridderinkhof et al., 2004; Desmet et al., 2011). If we assume that the activation in the DLPFC-dACC/medial FG was mainly triggered by response conflict, we should find the strongest brain activation in C2. Participants should be the least certain in C2, as the outcome was associated with the most variability compared to the other two conditions. There were two choices, none of which was more compelling than the other in this condition. However, the actual result was that the strongest brain activity in DLPFC-dACC/medial FG was found in C3 rather than C2. In contrast, if we assume that the conflict at the representational level was the main factor, we should find stronger activities in C3 than in the other two conditions. Our results were consistent with the latter assumption. It may be interpreted that regardless of the magnitude of the incongruent features, the dACC/medial FG simply detected whether the incongruent features (with/without) appeared, and then the incongruent features were forcefully inhibited or rejected by the DLPFC.

Our analysis also revealed a significant main effect of condition in the bilateral ventral anterior cingulate cortex (vACC) (BA32). Further analysis indicated greater negative activation in the bilateral vACC in C2 and C3 than in C1. Significant deactivation in the right vACC was observed in C3 compared to C2. Previous studies have suggested that the vACC may be a part of the DMN (Greicius et al., 2003; Hampson et al., 2006; Anticevic et al., 2012; Buckner and DiNicola, 2019). The DMN showed task-related decreased activity in cognitive tasks (e.g., working memory and classification tasks), and lower DMN activity on a trial-by-trial basis was associated with better cognitive performance (McKiernan et al., 2003; Mayer et al., 2010; Anticevic et al., 2012; Wei et al., 2013; Breukelaar et al., 2020). Task load and increasing task complexity were positively correlated with deactivation of the DMN (Wei et al., 2013; Hearne et al., 2015). For example, Mayer et al. (2010) used an easy or difficult visual search to encode one or three complex objects into WM (WM Load 1 and 3) and found that WM-load deactivations were predominantly located in the right medial PFC, medial parietal cortex, etc. Two other studies using the n-back task also found that DMN deactivation is modulated by increased cognitive load demand in healthy human subjects (Ceko et al., 2015; Finc et al., 2017). In this study, the WM load increased from C1 to C3 during categorization. There was a one-unit WM load difference between C1 and C2, and between C2 and C3. Accordingly, deactivation of the right vACC in the DMN significantly increased from C1 to C3. This finding showed that the suppression of the vACC (especially the right vACC) was sensitive to the level of cognitive effort depending on the WM load.

It is worth noting that the sample size in the current study is relatively small (*n* = 20). The number of participants was determined according to four previous studies (Chen et al., 2008; Garcin et al., 2012; Cai et al., 2014; Gao et al., 2016) in which the number of participants was 16, 20, 20, and 20. Considering the low reliability of fMRI activation (Elliott et al., 2020), future studies with more participants are needed to improve the reliability of our findings.

In conclusion, our study showed that activation of the DLPFC (BA9) and dACC/medial PFC (BA8/32) were associated with conflict control, and these brain activities depend on whether conflict was present rather than the magnitude of conflict during categorization. In contrast, the vACC (BA32), especially the right vACC, showed significant deactivation during categorization in the experimental conditions, consistent with the proposal that such task-induced deactivation within parts of the DMN depended on the specific characteristics of the WM load of the task. Our results provide additional information to understand the neural basis of cognitive control in the category induction.

## Data Availability Statement

The original contributions presented in the study are included in the article/supplementary material, further inquiries can be directed to the corresponding author/s.

## Ethics Statement

The studies involving human participants were reviewed and approved by the Research Ethics Committee of Southwest University. The patients/participants provided their written informed consent to participate in this study. Written informed consent was obtained from the individual(s) for the publication of any potentially identifiable images or data included in this article.

## Author Contributions

XC and YW conceived and designed the study. GL, QL, FX, and YZ collected data and performed the data analyses. XC wrote the manuscript. YW revised the manuscript and provided constructive discussions. All authors contributed to the article and approved the submitted version.

## Conflict of Interest

The authors declare that the research was conducted in the absence of any commercial or financial relationships that could be construed as a potential conflict of interest. The reviewer CL declared a shared affiliation, with one of the author YW to the handling editor at the time of the review.

## Publisher’s Note

All claims expressed in this article are solely those of the authors and do not necessarily represent those of their affiliated organizations, or those of the publisher, the editors and the reviewers. Any product that may be evaluated in this article, or claim that may be made by its manufacturer, is not guaranteed or endorsed by the publisher.
